# Supplementary material for: Regional mutational signature activities in cancer genomes
Source: PLoS Comput Biol. 2022 Dec 5;18(12):e1010733. doi: 10.1371/journal.pcbi.1010733 (PMC9754594; doi:10.1371/journal.pcbi.1010733)
Supplement: S1 Text — Abbreviations of tumor types analyzed in the study. Table B in S1 Text: Co-occurrence of changes in mutational signature activities and copy number aberrations, kataegis events, and TAD boundaries across 20 tumor types. For each tumor type, we conducted a randomization test to determine how many samples have a significant proportion of their changepoints overlapping with one of these features. Randomization tests were conducted by generating 10,000 random samples with the same number of changepoints as the original samples with changepoint locations randomized. Each random set of changepoints was compared to the original sample copy number profile, kataegis profile, or consensus TAD map to calculate the proportion of changepoints overlapping with a given feature. This set of proportions formed the null distribution against which the sample proportion was compared to determine a p-value (ɑ = 0.05). Table C in S1 Text: Distributions of genomic/epigenomic features compared between regions containing changepoints and upstream and downstream regions across 20 tumor types. Each column shows the proportion of changepoints in a cancer with significantly different genomic feature distributions between the region containing the changepoint and the upstream and/or downstream region. For each changepoint, feature distributions were compared between two equally-sized adjacent segments, one centered in the middle of the changepoint region and the other centered in the middle of the span between the changepoint and the nearest upstream/downstream changepoint or chromosomal boundary, if nearer. Distributions were compared using Fisher’s exact test for CNA, kataegis, and A/B compartment measurements, and using the Kolmogorov-Smirnov test for ATAC-Seq, DNAse-Seq, gene density, and replication timing measurements. For each sample, 10,000 randomly-located changepoints were generated and the same distributions were compared to form a null distribution of p-values. The proportion of null [file pcbi.1010733.s001.docx]

**Regional mutational signatures in cancer genomes** Caitlin Timmons, Quaid Morris, Caitlin F. Harrigan

**Table A in S1 Text:** Abbreviations of tumor types analyzed in the study.

| **Tumor type** | **Abbreviation** |
| --- | --- |
| Biliary adenocarcinoma | Biliary-AdenoCA |
| Bladder transitional cell carcinoma | Bladder-TCC |
| Bone osteosarcoma | Bone-Osteosarc |
| Breast adenocarcinoma | Breast-AdenoCA |
| Cervical cancer | Cervix |
| Glioblastoma | CNS-GBM |
| Colorectal adenocarcinoma | Colorect-AdenoCA |
| Esophageal adenocarcinoma | Eso-AdenoCA |
| Head and neck squamous cell carcinoma | Head-SCC |
| Kidney chromophobe renal cell carcinoma | Kidney-ChRCC |
| Kidney renal cell carcinoma | Kidney-RCC |
| Liver hepatocellular carcinoma | Liver-HCC |
| Lung adenocarcinoma | Lung-AdenoCA |
| Lung squamous cell carcinoma | Lung-SCC |
| Lymph B-cell non-Hodgkin lymphoma | Lymph-BNHL |
| Chronic lymphocytic leukemia | Lymph-CLL |
| Melanoma | Melanoma |
| Myeloproliferative neoplasm | Myeloid-MPN |
| Ovarian adenocarcinoma | Ovary-AdenoCA |
| Pancreatic adenocarcinoma | Panc-AdenoCA |
| Pancreatic neuroendocrine cancer | Panc-Endocrine |
| Prostate adenocarcinoma | Prost-AdenoCA |
| Stomach adenocarcinoma | Stomach-AdenoCA |
| Thyroid adenocarcinoma | Thy-AdenoCA |
| Uterine adenocarcinoma | Uterus-AdenoCA |

**Table B in S1 Text: Co-occurrence of changes in mutational signature activities and copy number aberrations, kataegis events, and TAD boundaries across 20 tumor types.** For each tumor type, we conducted a randomization test to determine how many samples have a significant proportion of their changepoints overlapping with one of these features. Randomization tests were conducted by generating 10,000 random samples with the same number of changepoints as the original samples with changepoint locations randomized. Each random set of changepoints was compared to the original sample copy number profile, kataegis profile, or consensus TAD map to calculate the proportion of changepoints overlapping with a given feature. This set of proportions formed the null distribution against which the sample proportion was compared to determine a p-value (ɑ = 0.05).

| **Tumor Type** | **Samples with significant changepoint-CNA overlap / N samples** | **Samples with significant changepoint-kataegis overlap / N samples** | **Samples with significant changepoint-TAD boundary overlap / N samples** |
| --- | --- | --- | --- |
| Melanoma | 0 / 107 | 0 / 107 | 21 / 107 |
| Lung-SCC | 0 / 48 | 1 / 48 | 9 / 48 |
| Eso-Adeno CA | 0 / 97 | 0 / 97 | 9 / 97 |
| Lung-Aden oCA | 0 / 33 | 0 / 33 | 0 / 33 |
| Colorect-A denoCA | 0 / 60 | 0 / 60 | 7 / 60 |
| Bladder-TC C | 1 / 23 | 0 / 23 | 3 / 23 |
| Stomach-A denoCA | 0 / 67 | 0 / 67 | 1 / 67 |
| Head-SCC | 0 / 54 | 0 / 54 | 0 / 54 |

| Lymph-BN HL | 0 / 106 | 4 / 106 | 1 / 106 |
| --- | --- | --- | --- |
| Uterus-Ade noCA | 0 / 51 | 0 / 51 | 6 / 51 |
| CNS-GBM | 0 / 41 | 0 / 41 | 2 / 41 |
| Kidney-RC C | 0 / 144 | 0 / 144 | 0 / 144 |
| Breast-Ade noCA | 0 / 194 | 1 / 194 | 0 / 194 |
| Panc-Aden oCA | 0 / 238 | 0 / 238 | 0 / 238 |
| Bone-Osteo sarc | 0 / 39 | 0 / 39 | 0 / 39 |
| Prost-Aden oCA | 0 / 145 | 1 / 145 | 0 / 145 |
| Lymph-CL L | 0 / 95 | 0 / 95 | 2 / 95 |
| Kidney-Ch RCC | 0 / 38 | 0 / 38 | 0 / 38 |
| Cervix-SC C | 0 / 20 | 0 / 20 | 0 / 20 |
| Thy-Adeno CA | 0 / 29 | 0 / 29 | 0 / 29 |

**Table C in S1 Text: Distributions of genomic/epigenomic features compared between regions containing changepoints and upstream and downstream regions across 20 tumor types.** Each column shows the proportion of changepoints in a cancer with significantly different genomic feature distributions between the region containing the changepoint and the upstream and/or downstream region. For each changepoint, feature distributions were compared between two equally-sized adjacent segments, one centered in the middle of the changepoint region and the other centered in the middle of the span between the changepoint and the nearest upstream/downstream changepoint or chromosomal boundary, if nearer. Distributions were compared using Fisher’s exact test for CNA, kataegis, and A/B compartment measurements, and using the Kolmogorov-Smirnov test for ATAC-Seq, DNAse-Seq, gene density, and replication timing measurements. For each sample, 10,000 randomly-located changepoints were generated and the same distributions were compared to form a null distribution of p-values. The proportion

of null distribution p-values less than or equal to the sample changepoint was recorded as the final significance measure (ɑ = 0.05). Cancer-specific DNAse-Seq measurements obtained from Polak et al. 2015 were available only for primary melanocytes and are thus not shown as a column in the table. We found that 77 / 786 melanoma changepoints have significantly different chromatin accessibility—as measured by DNAse-I accessibility index—between the region containing the changepoint and the upstream and/or downstream regions.

| **Tumor Type** | **Copy number aberrations** | **Kataegis events** | **Chromatin accessibility (A/B compartments)** | **Chromatin accessibility (ATAC-Seq)** | **Gene density** | **Replication timing** |
| --- | --- | --- | --- | --- | --- | --- |
| Melanoma | 68 / 786 | 71 / 786 | ND | 112 / 786 | 0 / 786 | 0 / 786 |
| Lung-SCC | 35 / 312 | 36 / 312 | 41 / 312 | 38 / 312 | 0 / 312 | 0 / 312 |
| Eso- AdenoCA | 21 / 259 | 6 / 259 | ND | 23 / 259 | 0 / 259 | 0 / 259 |
| Lung- AdenoCA | 0 / 10 | 0 / 10 | 0 / 10 | 2 / 10 | 0 / 10 | 0 / 10 |
| Colorect- AdenoCA | 45 / 558 | 9 / 558 | 172 / 558 | 61 / 558 | 0 / 558 | 0 / 558 |
| Bladder- TCC | 11 / 90 | 3 / 90 | 24 / 90 | 12 / 90 | 0 / 90 | 0 / 90 |
| Stomach- AdenoCA | 1 / 18 | 0 / 18 | ND | 1 / 18 | 0 / 18 | 0 / 18 |
| Head-SCC | 1 / 2 | 0 / 2 | ND | 0 / 2 | 0 / 2 | 0 / 2 |
| Lymph- BNHL | 3 / 80 | 20 / 80 | ND | ND | 0 / 80 | 0 / 80 |
| Uterus- AdenoCA | 47 / 371 | 0 / 371 | 67 / 371 | 54 / 371 | 0 / 371 | 0 / 371 |
| CNS- GBM | 5 / 146 | 15 / 146 | ND | 6 / 146 | 0 / 146 | 0 / 146 |
| Kidney- RCC | 0 / 11 | 0 / 11 | 3 / 11 | 1 / 11 | 0 / 11 | 0 / 11 |
| Breast- AdenoCA | 6 / 45 | 7 / 45 | 7 / 45 | 6 / 45 | 0 / 45 | 0 / 45 |
| Panc- | 1 / 26 | 3 / 26 | ND | ND | 0 / 26 | 0 / 26 |

| AdenoCA |  |  |  |  |  |  |
| --- | --- | --- | --- | --- | --- | --- |
| Bone- Osteosarc | 4 / 36 | 5 / 36 | ND | ND | 0 / 36 | 0 / 36 |
| Prost- AdenoCA | 2 / 9 | 1 / 9 | 3 / 9 | 0 / 9 | 0 / 9 | 0 / 9 |
| Lymph- CLL | 0 / 195 | 1 / 195 | ND | ND | 0 / 195 | 0 / 195 |
| Kidney- ChRCC | 1 / 20 | 0 / 20 | ND | ND | 0 / 20 | 0 / 20 |
| Cervix | 4 / 25 | 0 / 25 | ND | 1 / 25 | 0 / 25 | 0 / 25 |
| Thy- AdenoCA | 0 / 11 | 0 / 11 | ND | 0 / 11 | 0 / 11 | 0 / 11 |

**Table D in S1 Text: GenomeTrackSig changepoint recovery on original profiles compared to resampled profiles.** Analysis restricted to samples which display recurrent changepoints. As described in Methods, we run GenomeTrackSig with five bootstrap samples to create ‘original’ signature profiles. We run GenomeTrackSig with 20 bootstrap samples to create ‘resampled’ signature profiles. Matching resampled profiles to original profiles, we calculate the number of high-confidence changepoints (changepoints found in more than 1 bootstrap sample) recovered and the number of recurrent changepoints recovered. Within a cancer type, we compare all resampled profiles to all original profiles to calculate the overall number of recurrent changepoints in original profiles recovered by at least 7 resampled profiles.

| **Tumor Type** | **Samples with recurrent changepoints** | **Recurrent changepoints recovered** | **Samples with all recurrent changepoints recovered** | **High-confidenc e changepoints recovered** |
| --- | --- | --- | --- | --- |
| Melanoma | 67 / 107 | 22 / 22 | 47 / 67 | 377 / 401 |
| Lung-SCC | 20 / 48 | 2 / 2 | 20 / 48 | 116 / 116 |
| Eso-AdenoCA | 41 / 98 | 13 / 13 | 38 / 41 | 109 / 111 |
| Colorect-AdenoCA | 18 / 60 | 6 / 6 | 15 / 18 | 156 / 187 |
| Lymph-BNHL | 37 / 106 | 4 / 4 | 37 / 37 | 40 / 40 |
| Uterus-AdenoCA | 8 / 51 | 2 / 3 | 7 / 8 | 37 / 62 |
| CNS-GBM | 14 / 41 | 2 / 2 | 13 / 14 | 36 / 42 |
| Lymph-CLL | 57 / 95 | 12 / 12 | 57 / 57 | 69 / 69 |
