# Supplementary material for: Regional mutational signature activities in cancer genomes
Source: PLoS Comput Biol. 2022 Dec 5;18(12):e1010733. doi: 10.1371/journal.pcbi.1010733 (PMC9754594; doi:10.1371/journal.pcbi.1010733)
Supplement: S1 Note — (PDF) [file pcbi.1010733.s002.pdf]

Supplementary Note 1: Formal description of changepoint detection

Reproduced from Rubanova et al. [6]

A. Computing activity of mutational signatures. We represent each mutation as a K-dimensional binary vector "one-hot-encoding" of a mutation type where the k-th component is equal to 1, and other components are zeros. We will denote $\mathbf{x}^{(n)}$ to be the "one-hot-encoding of mutation n. A sample containing N mutations is represented as a $N \times K$ binary matrix $\mathbf{X}$, where each column corresponds one mutation.

$$\mathbf{x}^{(n)} = \begin{bmatrix} 0 \\ 0 \\ ... \\ 1 \\ ... \\ 0 \end{bmatrix}; \quad x_k^{(n)} = \begin{cases} 1, & \text{mutation } n \text{ belongs to type } k \\ 0, & \text{otherwise} \end{cases} \tag{1}$$

We will denote mutational signatures as K-dimensional probability vectors $\boldsymbol{\mu}_i$, where $i = \{1...M\}$ is an index over signatures. Signatures are fixed and are not updated during the training.
We aim to estimate signature activities $\boldsymbol{\pi}$ – the proportion of mutations generated by each signature.

We will use the following notation:
K – number of mutation types
M – number of signatures
N – number of mutations
$\mathbf{x}^{(n)}$ – K-dimensional binary vector of mutation n
$x_k^{(n)}$ – k-th component of vector $\mathbf{x}^{(n)}$
$\boldsymbol{\mu}_i$ – i-th signature (K-dimensional vector)
s_{ik} – k-th component of vector $\boldsymbol{\mu}_i$
$\boldsymbol{\pi}$ – signature activities (mixture coefficients, M-dimensional vector)
m_i – i-th component of $\boldsymbol{\pi}$ (signature activity of signature i)
z_n – signature assignment for mutation n

We represent mutation matrix $\mathbf{X}$ as a mixture of signature multinomials $\boldsymbol{\mu}_1, ..\boldsymbol{\mu}_K$ with mixture coefficients $\boldsymbol{\pi}$:

$$\mathbf{X} \sim \text{Multinomial}(N; \sum_{i=1}^{M} m_i \, \boldsymbol{\mu}_i) \tag{2}$$

We denote z_n to be the signature assignment of mutation n. The probabilities of mutation n to be assigned to i-th signature are equal to the mixing coefficients:

$$p(z_n = i|\boldsymbol{\pi}) = m_i; \quad i \in \{1..M\} \tag{3}$$

The probability of a mutation n to be generated by signature i is given by:

$$p(\mathbf{x}^{(n)}|z_n = i, \boldsymbol{\pi}, \boldsymbol{\mu}_1, ..\boldsymbol{\mu}_K) = \prod_{k=1}^{K} s_{ik}^{x_k^{(n)}} \; ; i \in \{1..M\}; n \in \{1..N\} \tag{4}$$

Then log likelihood of the collection of mutations in a sample:

$$\begin{gathered} \log L(\mathbf{X}|\boldsymbol{\pi}, \boldsymbol{\mu}_1, ..\boldsymbol{\mu}_K) = \sum_{n=1}^{N} \log p(\mathbf{x}^{(n)}|\boldsymbol{\pi}, \boldsymbol{\mu}_1, ..\boldsymbol{\mu}_K) = \\ \sum_{n=1}^{N} \log \sum_{i=1}^{M} p(\mathbf{x}^{(n)}|z_n = i, \boldsymbol{\pi}, \boldsymbol{\mu}_1, ..\boldsymbol{\mu}_K) p(z_n = i|\boldsymbol{\pi}) \end{gathered} \tag{5}$$

To estimate the activities, we fit mixing coefficients $\boldsymbol{\pi}$ in each bin using Expectation-Maximization (EM) algorithm Dempster et al. (1977). The EM algorithm iterates between updating a posterior distribution over z_n and updating an estimate of the mixing coefficients $\boldsymbol{\pi}$

We start with initializing EM algorithm with uniform mixing coefficients:

$$\pi_i^{(0)} = \frac{1}{M}; \quad i \in \{1..M\} \tag{6}$$

Then, we repeat the following E-step and M-step until the algorithm converges.
In E-step, at the t-th iteration, the posterior probabilities of mutation assignments to signatures are estimated as such:

$$p(z_n = i|\mathbf{x}^{(n)}, \boldsymbol{\pi}^{(t-1)}, \boldsymbol{\mu}_1, ..\boldsymbol{\mu}_K) = \pi_i^{(t-1)} \prod_{k=1}^{K} s_{ik}^{x_k^{(n)}}; \quad i \in \{1..M\}; \quad n \in \{1..N\} \tag{7}$$

In M-step we update the estimates of the mixing coefficients:

$$\pi_i^{(t)} = \frac{1}{N} \sum_{n=1}^{N} p(z_i|\mathbf{x}^{(n)}, \boldsymbol{\pi}^{(t-1)}, \boldsymbol{\mu}_1, ..\boldsymbol{\mu}_K); \quad i \in \{1..M\} \tag{8}$$

The algorithm has converged when the value of π is updated by less than 0.001 between iterations. The resulting mixture coefficients as the activities of the mutational signatures. We show the activities as percentage for the convenience of interpretation.

B. Pruned Exact Linear Time (PELT) Algorithm. We adapt Pruned Linear Exact Time (PELT) Killick et al. (2012) algorithm to detect change points in activity trajectories given cost function (likelihood) and BIC penalty. PELT is based on dynamic programming and uses heuristics to prune the set potential changepoints, thus reducing the computational time.
In this section, we will use the following notation:
T – number of genomic regions
P – number of changepoints
M – number of signatures

B.1. Locating change points. As previously described in the Methods section, we separate mutations into bins 100 mutations, each of which represents one genomic region. Our input is the set of mutation counts across 96 types for each genomic region: $y_{1:T} = (y_1, \ldots, y_T)$. We aim to find P changepoints, or in other words, $P+1$ segments. We denote $\tau_{1:P} = (\tau_1, \ldots, \tau_P)$ to be the boundaries for our segments, meaning each segment will contain the data points $y_{\tau_{i-1}}..y_{\tau_i}$.
Given a set of changepoints we can compute the likelihood of the data the following way. We fit mutational signatures within each segment (treating all mutations within each segment as one bin) and compute the likelihood $\hat{L}(y_{\tau_{i-1}}..y_{\tau_i})$ as described in A. The total likelihood is the sum of likelihoods in each segment:

$$\hat{L} = \sum_{i=1}^{P+1} L(y_{(\tau_{i-1}+1):\tau_i})$$

We aim to minimize the Bayesian Information Criterion (BIC):

$$\mathrm{BIC} = -2 \ln \hat{L} + k \cdot \ln(T)$$

where k is the number of parameters in our model and T is the number of genomic regions. In our case $k = (P+1) \cdot (M-1)$ as we fit $(M-1)$ signature activities in $(P+1)$ segments (recall that signature activities are probability vectors, and therefore sum to 1).
We adapt PELT objective to minimize the BIC criterion. PELT aims to minimize sum of cost functions at each time point, while using a penalty β for each placed changepoint

$$\text{minimize} \sum_{i=1}^{P+1} C(y_{(\tau_{i-1}+1):\tau_i}) + \beta(P+1)$$

Intuitively, we are trying to select changepoints which result in the lowest cost (or highest likelihood) while reducing the penalty associated with adding changepoints. We set the parameters as follows to make the PELT equivalent to BIC:

$$C = -2\hat{L}; \quad \beta = (M-1)\ln(T)$$

TrackSig-PELT algorithm finds the changepoints as follows. The algorithm starts with finding a partial solution in a subset of the genome and then increases the search space until changepoints are located over the whole genome. The algorithm keeps

track of the genomic regions $R_{\tau*}$ that satisfy the pruning condition and which will be considered as potential changepoints at further iterations. At each iteration τ^*, the algorithm considers adding a new changepoint out of the set of available genomic regions $R_{\tau*}$. To score a potential new changepoint, the algorithm refits the activities in bins formed by a potential changepoint. It finds a genomic region τ' with the smallest likelihood and adds it to the list of changepoints cp. Then the list of available genomic regions $R_{\tau*}$ is updated: the potential changepoints are removed from further consideration if the increase in likelihood associated with this changepoint does not exceed the complexity penalty β.

B.2. Pruning. PELT provides an improvement in runtime by pruning certain changepoints from consideration. We prune genomic region t if for all $t < s < T$:

$$C(y_{(t+1):s}) + C(y_{(s+1):T}) + \beta \leq C(y_{(t+1):T}) \tag{9}$$

The cost of placing the last changepoint prior to T at t will always be higher than cost of placing the last changepoint prior to T at s. Given this result, we can eliminate t as a potential changepoint for all iterations of the dynamic programming algorithm as it will never be optimal going forwards.

Algorithm 1 TrackSig PELT Method (Killack and Eckley 2012)

Input: Mutation counts at each genomic region $(y_1, y_2, ..y_T)$

Initialize: Set $\beta = (M-1)\ln(T)$; $F(0) = 0$; cp = {}; $R_1 = \{0\}$
for $\tau^* = 1, ..., T$ **do**
    Calculate $F(\tau*) = \min_{\tau \in R_{\tau^*}} [F(\tau) + C(y_{(\tau+1):\tau^*}) + \beta]$, where $C(y_{(\tau+1):\tau^*}) = -2\hat{L}(y_{(\tau+1):\tau^*})$
    Let $\tau' = \arg\min_{\tau \in R_{\tau^*}} [F(\tau) + C(y_{(\tau+1):\tau^*}) + \beta]$
    Append τ' to cp
    Set $R_{\tau^*+1} = \{\tau \in R_{\tau^*} \cup \{\tau*\} : F(\tau) + C(y_{\tau+1:\tau^*}) + \beta \leq F(\tau*)\}$
end for
return cp – a set of changepoints
