# Supplementary figures and images for: Regional mutational signature activities in cancer genomes
Source: PLoS Comput Biol. 2022 Dec 5;18(12):e1010733. doi: 10.1371/journal.pcbi.1010733 (PMC9754594; doi:10.1371/journal.pcbi.1010733)

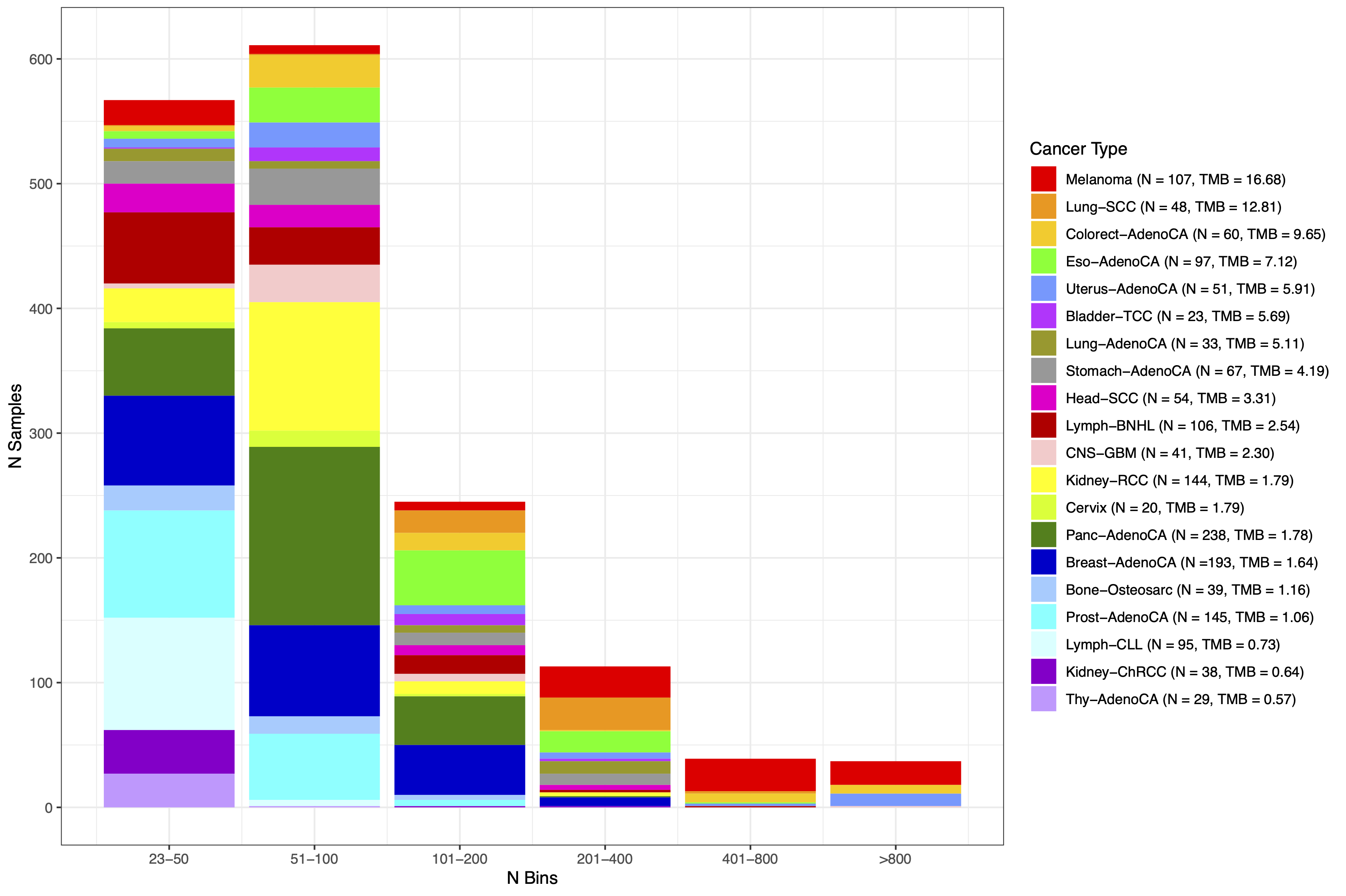

Supplement: S1 Fig — Stacked bar chart depicting the distribution of bin numbers across samples, colored by cancer type. Minimum number of bins is 23 (Melanoma, Lung-AdenoCA, Lymph-BNHL, Kidney-RCC, Prost-AdenoCA, Lymph-CLL, Kidney-ChRCC, Thy-AdenoCA) and maximum number of bins is 2895 (Colorect-AdenoCA). Sample size and geometric mean TMB is shown for each cancer type. (TIFF) [file pcbi.1010733.s003.tiff]

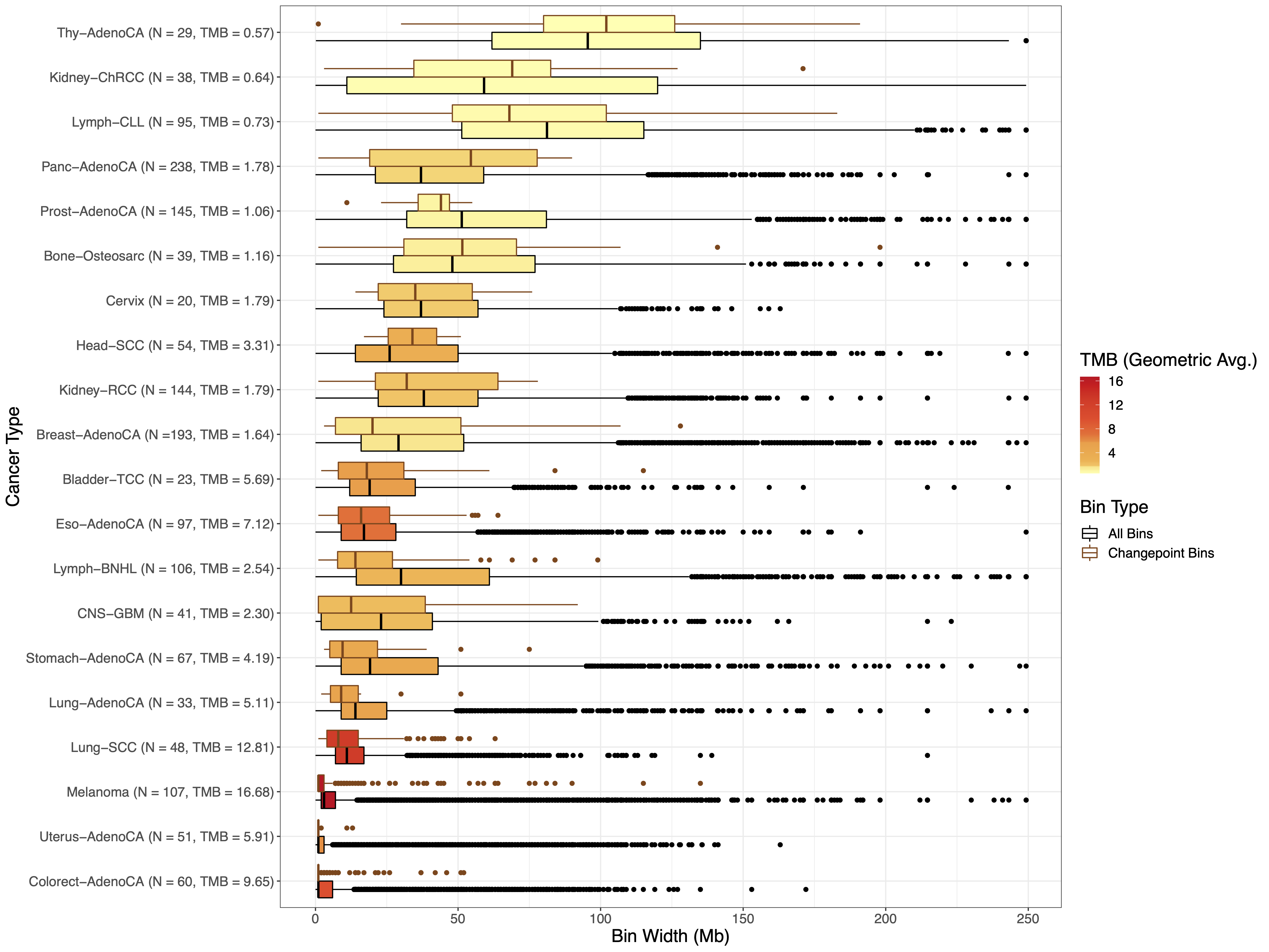

Supplement: S2 Fig — Boxplots showing the range of bin widths, in megabases, for all samples analyzed in the study. Boxplots are outlined according to the type of bin plotted, either all bins or bins containing changepoints. Sample size and geometric mean TMB is shown for each cancer type. Boxplots are colored according to geometric mean TMB. (TIFF) [file pcbi.1010733.s004.tiff]

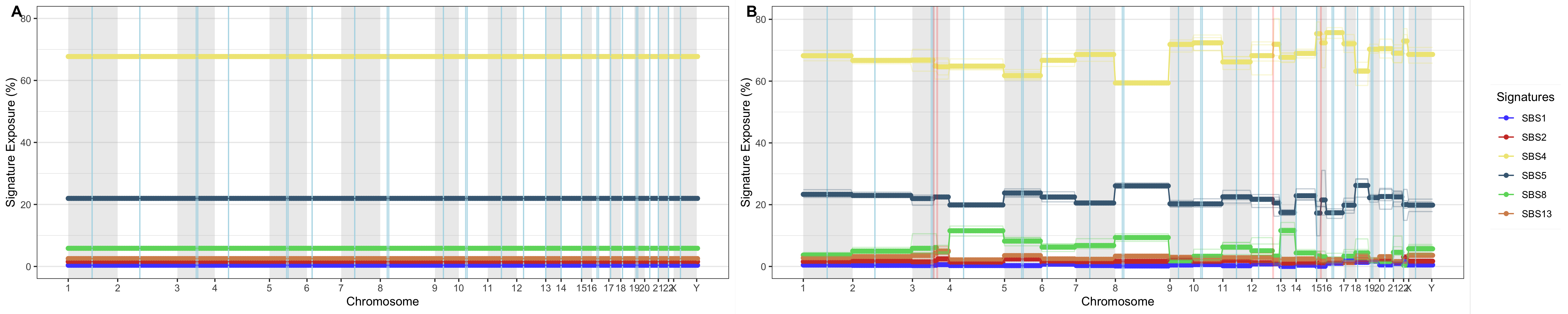

Supplement: S3 Fig — Input data is a Lung-SCC genome with 78,839 mutations. A bin size of 200 mutations was used and 5 bootstraps were performed for each experiment. Each point is a signature activity estimate at one bin of mutations. Alternating gray and white bars distinguish chromosomes and blue vertical lines show centromere positions. Red vertical lines denote changepoints, and the opacity of changepoints represents their bootstrap support. (TIFF) [file pcbi.1010733.s005.tiff]

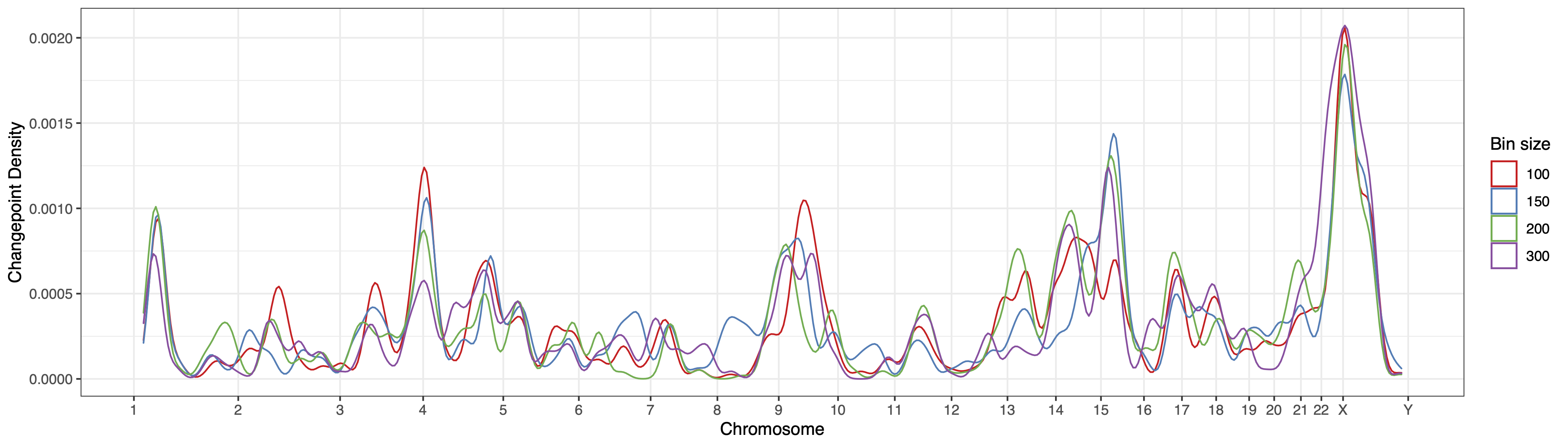

Supplement: S4 Fig — GenomeTrackSig was run genome-wise on 32 Lung-SCC samples with a bin size of either 100, 150, 200, or 300. Density plot across the genome of pooled changepoint positions in all samples is shown for each bin size analyzed. (TIFF) [file pcbi.1010733.s006.tiff]

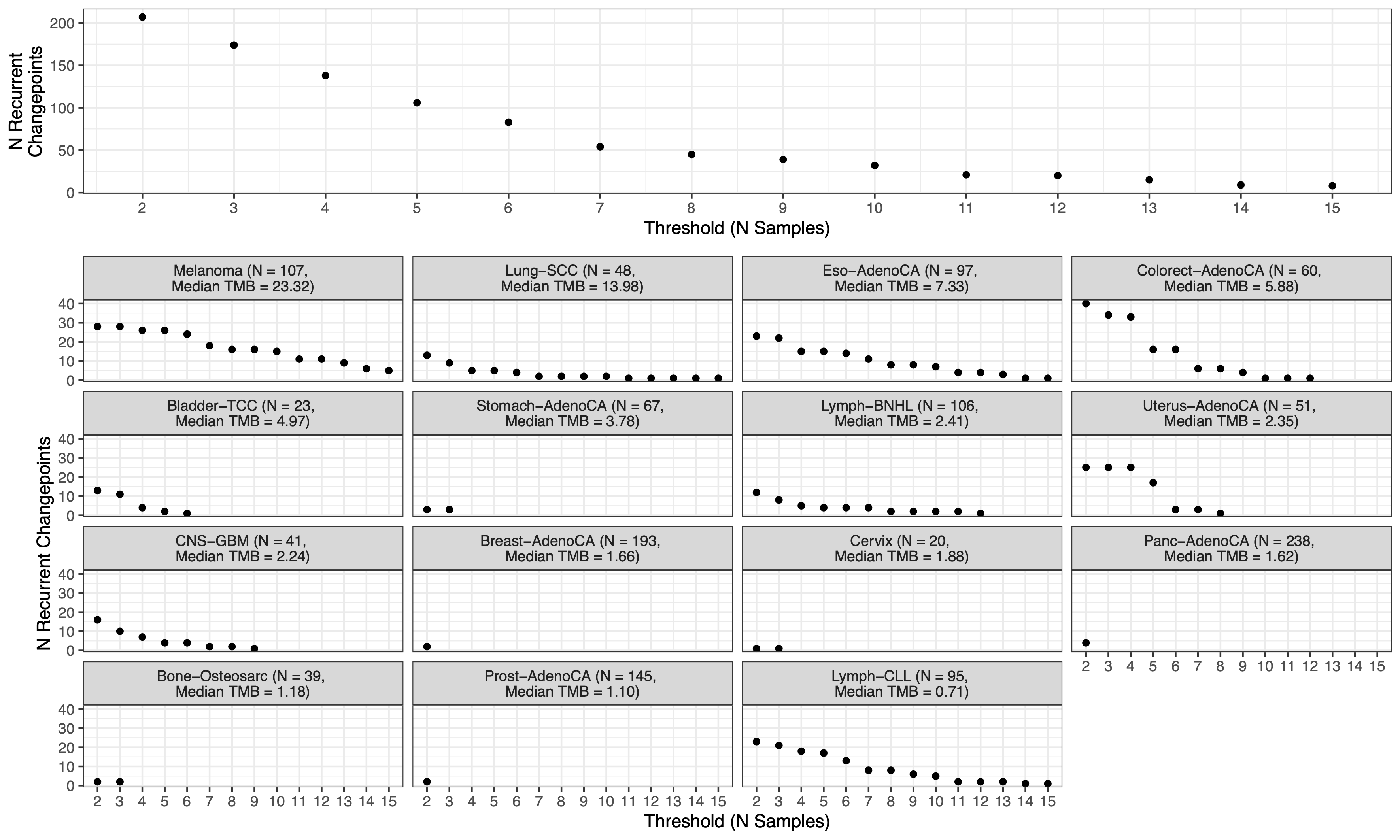

Supplement: S5 Fig — Top: Number of recurrent changepoints identified across all cancer types depending on which number of samples is used as the threshold to determine which changepoints are considered ‘recurrent.’ Bottom: Number of recurrent changepoints identified in each cancer type compared to the sample threshold. Sample size and median tumor mutational burden is shown for each cancer type. (TIFF) [file pcbi.1010733.s007.tiff]
