# Supplementary material for: Regional mutational signature activities in cancer genomes
Source: PLoS Comput Biol. 2022 Dec 5;18(12):e1010733. doi: 10.1371/journal.pcbi.1010733 (PMC9754594; doi:10.1371/journal.pcbi.1010733)
Supplement: Attachment — Submitted filename: Timmons_GenomeTrackSig_PLOSCB_Reviewer_Comments.docx [file pcbi.1010733.s008.docx]

October 31, 2022

Simone Zaccaria

Computational Cancer Genomics Lab

University College London Cancer Institute

Dear Dr. Zaccaria,

Thank you for the opportunity to revise our manuscript entitled “Regional mutational signature activities in cancer genomes.”. We appreciate the careful review and constructive suggestions. We have revised the manuscript according to the editor’s and reviewers’ comments. In particular, we have added additional simulations to demonstrate the robustness of our method in recovering mutational signature activity changes. We have also substantially expanded the Methods section to include a formal description of the GenomeTrackSig algorithm and descriptions of the simulation and randomization testing methods used. We believe that the manuscript is substantially improved after making the suggested edits.

Following this letter you will find the editor and reviewer comments along with our responses highlighted in color. The revision has been completed in consultation with all co-authors, and each author has given approval to the final form of the revision. We thank you for your consideration of our revised manuscript.

Sincerely,

Cait Harrigan

**Editor Comments:**

*We sincerely apologise for the delay in this review process. We have now finally received the comments from three reviewers and, based on their recommendations, we would be glad to invite a minor revision of your manuscript to be considered for publication. The reviewers agree on the importance and novelty of the work, but they also believe that a few minor comments should be addressed in a final version of your manuscript before final acceptance. Particularly, based on reviewers' comments, we would like to ask the authors to address two of the main remaining comments raised by the reviewers:*

*1- Could the authors add a minimal set of basic simulations, possibly comparing with the previous method GenomeTrackSig, to show the accuracy of your method?*

*2- Could the authors revise the text according to reviewers' recommendations, in particular adding a complete description of the method?*

Thank you for this helpful feedback. As described more thoroughly in our point-by-point responses, we have added two sets of simulations to the manuscript. One simulation demonstrates the novelty of GenomeTrackSig compared to the original TrackSig algorithm by analyzing the evolutionary timing (computed by TrackSig as inferred cancer cell fraction) of mutations within each bin of mutations that GenomeTrackSig aggregates by chromosomal location. We find that there are almost no genomic regions that exclusively contain mutations from one CCF segment, which demonstrates the novelty of our method since the changepoints identified by GenomeTrackSig could not be found using TrackSig. Additionally, we demonstrate the robustness of GenomeTrackSig’s changepoint placement to resampling of the mutation types, and find that the majority of changepoints are recovered. Furthermore, we have extensively revised the Methods section to include a more formal description of the algorithm, description of GenomeTrackSig parameters, the simulation approaches described above, and the statistical methods used to test for associations between changepoints and other genomic features.

Besides these main points, we have also revised the Introduction to eliminate dependence on TrackSig and make it accessible to a broader audience, and we have extensively expanded the package documentation and addressed the data availability issues raised by Reviewer #3.

**Reviewer #1 Comments:**

*The authors have added additional details and analyses that make the results in this revised version of the manuscript even more interesting, and they have successfully addressed several reviewers’ comments.*

Thank-you for your kind words.

*There are however some key comments that have not been appropriately addressed, especially related to the new algorithm, its benchmarking, and formal descriptions of the formal methods. Formal descriptions of methods are essential for both assessing and demonstrating the robustness of the proposed analysis and results, as well as for reproducibility. Therefore, these remaining comments should be appropriately addressed before the manuscript could be considered for final publication.*

With the lack of page limits, we have now been able to add a formal description of the algorithm and a benchmarking section. See below for details.

*1. No simulation nor benchmarking analysis has been performed to assess and demonstrate the performance of GenomeTrackSig, as suggested by previous reviewers. While the authors highlight that the focus of the manuscript is on the outcome of the analysis, the results of the analysis cannot be believed if the accuracy and robustness of the proposed method are not clearly demonstrated. I would recommend the authors to consider using simulations to demonstrate the performance of the algorithm, as well as to address other reviewers’ comments, such as the impact of segment size, mutational burden, etc.*

We discuss our efforts to evaluate the impact of bin size in the Methods and second paragraph of the Results section, and display our findings in Fig. S4. The key elements of the text are reproduced below for convenience:

“GenomeTrackSig includes flexible options for analyzing samples, including choice of bin size, segmentation approach (genome-wide or chromosome-wise), and bootstrap resampling method. By default, we partition the set of mutations in a genome into bins of 100 mutations each, where the size of the chromosome region (i.e., number of basepairs) spanned by a bin will vary depending on mutation density. We require each segment in the segmentation solution to include at least one bin.”

“GenomeTrackSig allows flexibility in the choice of bin size, and we find that changepoint placement is relatively insensitive to bin sizes greater than the recommended minimum of 100 mutations (Fig. S4).”

We have now added simulations where the segment boundaries are provided and evaluate the robustness of GenomeTrackSig’s changepoint placement to resampling of the mutation types. We have added additional text in the Methods and Results section and an additional supplementary table (reproduced below) to describe these analyses. Overall, we find that the vast majority of robust changepoints in our original profiles are robustly identified in simulated profiles. All but one recurrent changepoints are also recurrent across resampled profiles, and the vast majority of resampled profiles (with the possible exceptions of melanoma and uterine cancer) recover the same recurrent changepoints as their original counterparts. While we restrict this analysis to samples with recurrent changepoints (since these are the focus of our Results), our findings are consistent across samples with low and high mutational burden.

Table S4: GenomeTrackSig changepoint recovery on original profiles compared to resampled profiles. Analysis restricted to samples which display recurrent changepoints. As described in Methods, we run GenomeTrackSig with five bootstrap samples to create ‘original’ signature profiles. We run GenomeTrackSig with 20 bootstrap samples to create ‘resampled’ signature profiles. Within a cancer type, we compare all resampled profiles to all original profiles to calculate the overall number of recurrent changepoints in original profiles recovered by at least 7 resampled profiles. Matching resampled profiles to original profiles, we calculate the number of recurrent changepoints recovered and the number of high-confidence changepoints (changepoints found in more than 1 bootstrap sample) recovered.

| **Tumor Type** | **Samples with recurrent changepoints** | **Recurrent changepoints recovered** | **Samples with all recurrent changepoints recovered** | **High-confidence changepoints recovered** |
| --- | --- | --- | --- | --- |
| Melanoma | 67 / 107 | 22 / 22 | 47 / 67 | 377 / 401 |
| Lung-SCC | 20 / 48 | 2 / 2 | 20 / 20 | 116 / 116 |
| Eso-AdenoCA | 41 / 98 | 13 / 13 | 38 / 41 | 109 / 111 |
| Colorect-AdenoCA | 18 / 60 | 6 / 6 | 15 / 18 | 156 / 187 |
| Lymph-BNHL | 37 / 106 | 4 / 4 | 37 / 37 | 40 / 40 |
| Uterus-AdenoCA | 8 / 51 | 2 / 3 | 7 / 8 | 37 / 62 |
| CNS-GBM | 14 / 41 | 2 / 2 | 13 / 14 | 36 / 42 |
| Lymph-CLL | 57 / 95 | 12 / 12 | 57 / 57 | 69 / 69 |

*2. Related to the previous point and other reviewers’ comments, it would also be important to benchmark the results of GenomeTrackSig with those of previous methods. In particular, it would be important to demonstrate that the results obtained by GenomeTrackSig could have not been obtained in a straightforward way by simply using TrackSig (ie. without the new segmentation introduced by GenomeTrackSig).*

TrackSig segments on the CCF-axis rather than location-axis. To try to reproduce our results with TrackSig, we classified each mutation in a location bin according to its TrackSig clone assignment. We calculate the number of distinct clone assignments within a bin, and then call a changepoint whenever the identity of the clone with the highest number of mutations changes. We check for agreement between GenomeTrackSig changepoints and changepoints based on clone assignments, and we find that 75% of samples have no agreement between the position-based changepoints (GenomeTrackSig) and the CCF-based changepoints (TrackSig), showing that, as expected, the changepoints we find with GenomeTrackSig could not be found without the mutation binning modification that GenomeTrackSig introduces. We have added a subsection to the Methods called “Comparing position-based binning to CCF-based binning” and text to the Results section “Many changepoints are characterized by signatures associated with subclonal expansion” describing this finding.

*Moreover, while the authors have reported some differences with the previous method SigMa and have added a useful comparison section, these differences do not prevent the possibility to compare the basic performance of the two methods (GenomeTrackSig and SigMa) in estimating the spatial genomic distributions of the identified mutational signatures. Thus, comparing the performance of those methods using the proposed simulations would be important to demonstrate the impact of the new contribution in this manuscript.*

We struggled to compare our method to SigMa because it requires input data in an unspecified JSON format, and the links to example data provided appear to be broken. We found that there is an open issue reported on the SigMa Github page dated February 2021 asking for example data, but this has not been resolved. We have contacted the authors of SigMa via email, but we did not receive a reply after two weeks. Since we were unable to successfully run the SigMa method, we have moved our discussion of SigMa to the Introduction section of the manuscript where we describe related methods to GenomeTrackSig, and do not include it as part of our results.

*3. The authors have introduced a new method section to better explain the new proposed algorithm. However, this section is still lacking formal mathematical descriptions; especially the entire section does not include a single equation to describe the proposed model and the relationship between the defined variables. For example, what is the probabilistic generative model on which the EM algorithm has been applied to (definition of random variables, related probabilities, etc.), or what is the relation linking the mutation matrix to the signature activities? Also, formal definitions are missing: what is the mathematical definition, domain and constraints of variables s_i? What is the definition of mutation type (I guess nucleotide context?)? What are the exact variables and equation used for applying the BIC criterion? What is the optimization function that defines an optimal segmentation? Etc.*

With the expanded page limit restriction, we are now able to add this section. We have added some descriptive text to the methods, and reproduced the formal model description of TrackSig-PELT as a supplemental note.

*4. The current structure of the manuscript is confusing as the Result section describes the results of the analysis even before explaining or describing the new algorithm that have been developed to perform these analyses. Similarly to other manuscripts in this journal, it would be beneficial to start Results with a paragraph dedicated to providing a summary of the new proposed method.*

We thank the reviewer for this suggestion to improve our Results section. We have added a paragraph at the beginning of this section which introduces GenomeTrackSig and summarizes the key elements of the method. Here, we also describe the results of our new analysis which evaluates the robustness of the changepoints we identify using GenomeTrackSig.

*5. Method section is substantially lacking several details and required descriptions, for example a full description of the processing pipelines applied to prepare the data and perform the analysis. Even more importantly, the formal descriptions of the sophisticated statistical analyses reported in the reviewers’ response (randomizations, generation of null distribution for change points, bootstrapping, etc.) are not reported in the Method section. I would invite the authors to carefully add in this section the formal details and descriptions of all the performed analyses, as well as the data processing and the new proposed algorithm.*

We thank the reviewer for their recommendations to improve the Methods section. We have added descriptions of the bootstrapping strategies supported by GenomeTrackSig within our description of the different ways that users can implement the method. We have also included descriptions of our simulations to evaluate the robustness of GenomeTrackSig’s changepoint placement given the segment boundaries within a sample, as mentioned in an earlier comment. Additionally, we provide more extensive descriptions of the randomization methods used to test for associations between changepoints and different genomic features within the Methods section.

**Reviewer #2 Comments:**

*I was a reviewer for RECOMB-CCB. The authors have properly addressed all reviewers’ comments, providing additional experiments and evidence supporting their main conclusions. The new version of the manuscript greatly improves over the previous version.*

*I have only a minor comment. The "Related work" section describes is 1 paragraph describing the related method SigMa, and coming after the Results section it feels a bit out of place (since the method is not described as a result). I think it makes more sense to incorporate such paragraph in the Introduction or in the Materials and Methods section.*

We thank the reviewer for this suggestion. We have added additional descriptive text to the Introduction section on related methods, and incorporated the section on SigMa there.

**Reviewer #3 Comments:**

*One of my students has previously helped with reviewing this paper for conference and she informed me that all of her comments are now addressed. The paper was presented at RECOMB-CCB earlier this year in San Diego and we attended the interesting talk given by the first author. Considering that this is a journal version, I would kindly ask the authors to do the following:*

*1. The paper would benefit from a substantial revision and extension of the presentation. This primarily refers to extending Introduction and Methods sections to make them more accessible to the broader audience.*

*Dependence on TrackSig and other previous works should be minimal. Some descriptions can be moved to Supplementary if needed. I totally understand that this was difficult to do previously for the conference submission due to page limits, deadlines etc. and this was to some extent acceptable due to very specialized audience at the conference, but I am concerned that understanding the paper might be challenging for less specialized readers, which could limit its potential impact.*

This is a great point! We have now rewritten the manuscript for the PLOS Computational Biology audience, and substantially edited the Introduction. There, we describe TrackSig and TrackSigFreq in more detail so readers can better understand the contributions of those methods and their key differences with GenomeTrackSig. We have also extensively expanded the Methods section, as described in our responses to Reviewer #1.

*2. The documentation at the github repository (https://github.com/morrislab/GenomeTrackSig) is currently incomplete. To give a simple example, no explanation of the input files is provided.*

*Please provide more detailed and complete documentation for everything. Also, I recommend you to provide (in README) details of who should be preferably contacted and at what email address in case that a potential user has some questions, but I leave this as optional considering that some contact details will be provided in the main paper.*

Thank you for these recommendations. We have updated the [R package GitHub repository](https://github.com/morrislab/GenomeTrackSig#additional-genometracksig-options) so that we describe the input datasets in more depth and we have added contact details for users who have questions. We have also substantially extended the package demo beyond the basic use case. We describe how users can implement additional visualizations such as plotting contextual genomic features alongside signature profiles, and how they can implement additional package options which include bootstrapping and strategies to reduce runtime via chromosome-level segmentation and parallelization. In addition, we have also created a supplementary [Analysis GitHub repository](https://github.com/ctimmons1/GenomeTrackSig-supplement) to store relevant data processing, analysis, and visualization code used for analyses in this paper.

*3. In addition to the previous point, I noticed that there is file Example_cna.txt in extdata folder (https://github.com/morrislab/GenomeTrackSig/blob/master/inst/extdata/Example_cna.txt). While it is not clear from the documentation what purpose this file serves and whether it is needed at all (I do not see it being used in any of the example commands shown in README so it is possible that this is something that the authors forgot to delete from the repository), its last column seems to be consisting only of 2's. If this is true and the file is a part of the input/output, then this suggests that the provided examples can perhaps be improved to cover a wider range of permissible inputs. Please double check this.*

Thank you for pointing this out. It is indeed a file that we forgot to delete. We have now fixed it and double-checked that only relevant files are included on the GitHub page.
